# ‘Climate Healing Stones’: Common Minerals Offer Substantial Climate Change Mitigation Potential

**DOI:** 10.1007/s00267-024-01945-x

**Published:** 2024-02-19

**Authors:** Chris Pratt, Zainab Mahdi, Ali El Hanandeh

**Affiliations:** 1 School of Environment and Science, Australian Rivers Institute, Griffith University, Kessels Road, Nathan, QLD 4111 Australia; 2 School of Engineering and Built Environment, Australian Rivers Institute, Griffith University, Kessels Road, Nathan, QLD 4111 Australia; 3 School of Engineering and Built Environment, Griffith University, Kessels Road, Nathan, QLD 4111 Australia

**Keywords:** Carbon dioxide, Climate change, Geosphere, Greenhouse gases, Minerals

## Abstract

This review proposes that mineral-based greenhouse gas (GHG) mitigation could be developed into a substantial climate change abatement tool. This proposal was evaluated via three objectives: (1) synthesise literature studies documenting the effectiveness of geological minerals at mitigating GHG emissions; (2) quantify, via meta-analysis, GHG magnitudes that could be abated by minerals factoring-in the carbon footprint of the approach; and (3) estimate the global availability of relevant minerals. Several minerals have been effectively harnessed across multiple sectors—including agriculture, waste management and coal mining—to mitigate carbon dioxide/CO_2_ (e.g., olivine), methane/CH_4_ (e.g., allophane, gypsum) and nitrous oxide/N_2_O (e.g., vermiculite) emissions. High surface area minerals offer substantial promise to protect soil carbon, albeit their potential impact here is difficult to quantify. Although mineral-based N_2_O reduction strategies can achieve gross emission reduction, their application generates a net carbon emission due to prohibitively large mineral quantities needed. By contrast, mineral-based technologies could abate ~9% and 11% of global CO_2_ and CH_4_ anthropogenic emissions, respectively. These estimates conservatively only consider options which offer additional benefits to climate change mitigation (e.g., nutrient supply to agricultural landscapes, and safety controls in landfill operations). This multi-benefit aspect is important due to the reluctance to invest in stand-alone GHG mitigation technologies. Minerals that exhibit high GHG mitigation potential are globally abundant. However, their application towards a dedicated global GHG mitigation initiative would entail significant escalation of their current production rates. A detailed cost-benefit analysis and environmental and social footprint assessment is needed to ascertain the strategy’s scale-up potential.

## Introduction

More than 5000 naturally-occurring minerals have been identified (Whitney and Evans 2010). Aside from their obvious usefulness as economically relevant metals, naturally-occurring minerals have a wide range of applications from food preservation (Henriet et al. 2014) to construction (Charola et al. 2007) to electronics manufacturing (Wu et al. 2016). An emerging area of mineral application is the field of climate change mitigation. Recently, mineral-based technologies and management approaches have been harnessed to mitigate emissions of the ‘big three’ direct greenhouse gases (GHGs) (i.e., carbon dioxide, methane and nitrous oxide), as well two key indirect emission sources—(1) carbon from warming soils and (2) ammonia volatilisation (which leads to secondary N_2_O emissions).

In the past 15 years, researchers have investigated a broad suite of mineral-based technologies for their potential to mitigate GHG emissions across varying human-modified and natural environments. These settings include: agricultural cropping landscapes (Zaman and Nguyen 2010; Pratt et al. 2016); wastewater treatment systems (Pangala et al. 2010); as well as marine (Köhler et al. 2010) and atmospheric (Taylor et al. 2016) sinks of CO_2_. The types of minerals used in these endeavours encompass an exceptionally diverse range, representing more than half of the major mineral family groups—i.e., the silicates (both primary and secondary), the oxides and hydroxides, the sulfates, and the carbonates. Moreover, these mineral families are ubiquitously distributed which is an important factor when considering their incorporation into mitigation technologies.

The emergence of mineral-based GHG mitigation technologies is timely, as solutions for climate change mitigation are being rapidly sought (Ireland and Clausen 2019). Indeed, Brauch et al. (2019) note that an ‘unprecedented’ shift towards low-carbon technologies will be needed to meet 2030 global temperature targets. Here, we identify an opportunity to support this challenge through reviewing the state of knowledge on mineral-based GHG mitigation technologies, highlighting areas of promise and the potential impact that these technologies can offer.

Specific aims of this study are to: (1) synthesise the effectiveness of mineral-based technologies for mitigating anthropogenic GHG emissions; (2) evaluate global abundances of the key minerals used in GHG mitigation technologies using a semi-quantitative ranking approach; and (3) quantify the proportion of anthropogenic emissions that mineral-based mitigation technologies can achieve, factoring-in carbon footprint and return-on-investment considerations. This final aim is particularly important, because there is a general reluctance to invest in stand-alone GHG abatement technologies (Pratt and Tate 2018). Highlighting the additional benefits to climate change mitigation that mineral-based abatement technologies can offer will hopefully enhance the attractiveness of the approach and help develop it as a substantial tool in global efforts to combat climate change. This review focuses on terrestrial applications of mineral-based abatement technologies. We extend the scope of our work across the full suite of minerals as defined in geological terms—i.e., naturally-occurring crystalline solids free of organic carbon. However, we also include two important technical exceptions that have been pursued in GHG abatement research: (1) synthetic zeolites, which have wide use in industrial processes; and (2) lignite—a type of coal that has been explored in nitrogen GHG mitigation.

## Analytical Framework

Estimates of key GHG emission magnitudes were developed using the most recent Intergovernmental Panel on Climate Change (IPCC) reporting data in conjunction with scientific literature and databases. Climate change mitigation effectiveness was quantified by averaging upper and lower estimates reported in scientific literature sources, factoring-in Life Cycle Assessment metrics—covering extraction, processing and transport emissions—reported by Moosdorf et al. (2014). Estimates of reserves and production rates of key geological minerals possessing climate change mitigation potential were compiled predominantly from US Geological Survey databases as well as other scientific sources.

## Overview of Anthropogenic GHG Estimates

Total annual anthropogenic GHG emissions were 52 Pg CO_2_-e about a decade ago, according to the most recent (5th) IPCC synthesis report (IPCC 2014)—the sixth synthesis report (2023) estimates the GHG emission has reached 59 Pg CO_2_-e in 2019, an increase of 14% over the previous reporting period (IPCC 2023). Ritchie et al. (2020), for example, report a CO_2_ estimate of 35 Pg for the year 2020, which is exactly the same as recorded for 2014, while the IEA (2021) notes an annual anthropogenic CH_4_ emission of 9.5 Pg CO_2_-e in 2020, compared with 9 Pg in 2014 (IPCC 2014).

**Fig. 1:**
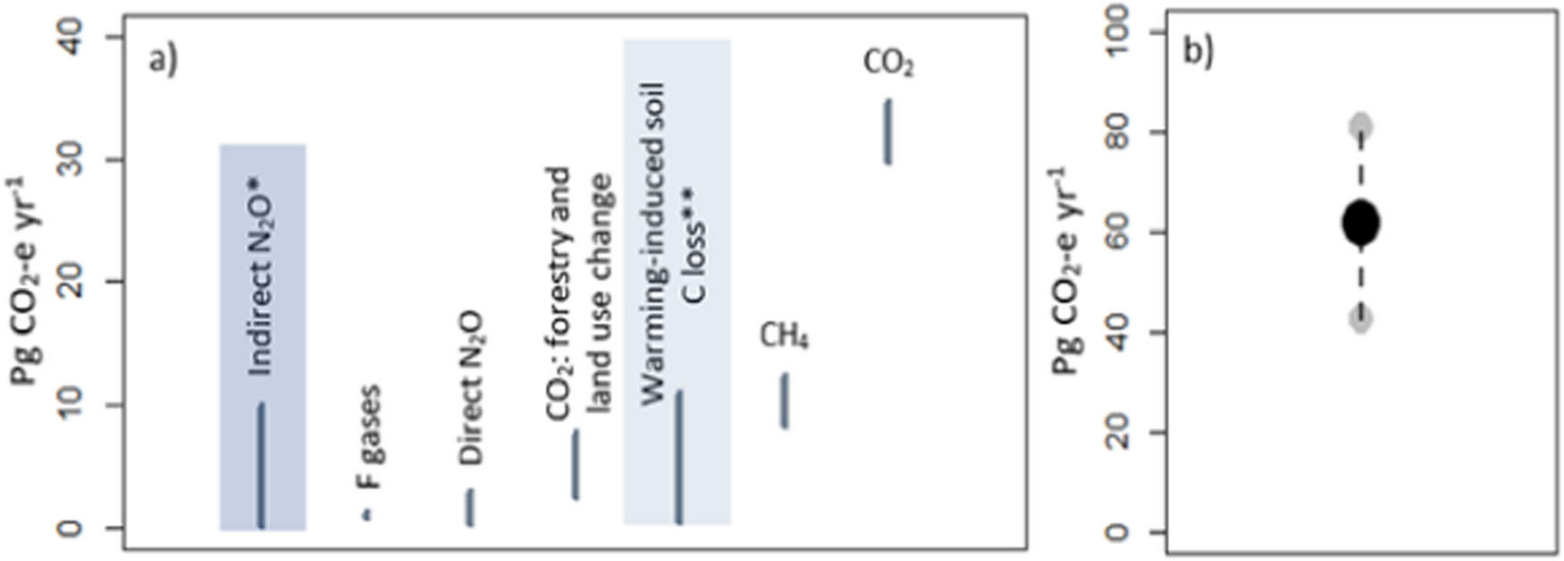
**a** Global emission ranges (lower and upper) estimates for the principal anthropogenic greenhouse gases, updated from IPCC (2014), with additional key GHGs highlighted: *indirect N_2_O from NH_3_ volatilisation, from Turner et al. (2015); and **CO_2_ derived from increased soil respiration due to projected warming, from Crowther et al. (2016); **b** lower, mean and upper total annual anthropogenic GHG emissions, synthesised from IPCC (2014), Turner et al. (2015) and Crowther et al. (2016)

The major GHGs are, in order of magnitude, CO_2_, CH_4_, N_2_O and the fluorinated gases (CFC gases). Methane’s contribution to total emissions has recently been reported to be dependent on its origin, with a modified Global Warming Potential from 27.2 (non-fossil) to 29.8 (fossil) adopted by the IPCC, AR6 report (IPCC 2021). In addition, hydroelectric dams are becoming recognised as a significant CH_4_ emission source following a series of publications reporting on their impact (Bambace et al. 2007, Lima et al. 2008). Figure 1a shows emission magnitudes and uncertainties for the major GHGs and Fig. 1b presents cumulative anthropogenic GHG emissions. Two additional important GHG classes are included in Fig. 1: (1) indirect N_2_O emissions from ammonia volatilisation, and (2) soil CO_2_ emissions estimated to occur resulting from warming climates. Although indirect N_2_O emissions are included as part of the total N_2_O emission estimate in the 5th IPCC assessment report, their relative contribution is not quantified in that document. Moreover, research by Turner et al. (2015) suggests that the upper estimate of indirect N_2_O emissions (Fig. 1) could be much higher than currently documented by IPCC reporting.

Warming-induced soil CO_2_ emissions are an example of a feedback response predicted to occur in a warming world. These types of sources—which also include increased CH_4_ emissions from permafrost thawing (Walter Anthony et al. 2016; Neumann et al. 2019)—are becoming increasingly recognised as important contributors to total anthropogenic GHG budgets.

Note that consideration of warming-induced soil CO_2_ and new estimates of indirect N_2_O emissions results in a higher total anthropogenic GHG production value (Fig. 1b) than reported by the IPCC (2014) and other more recent estimates (Crippa et al. 2021), by ~10 Pg CO_2_-e year^−1^.

Appraisal of the latest emission trends suggest that anthropogenic emission profiles will continue to be dominated by CO_2_, produced both directly and through indirect processes (Fig. 1). Yet, contributions from CH_4_ and N_2_O are also projected to be considerable—ranging between 14% and 43% of total anthropogenic GHG production according to the IPCC and literature estimates presented in Fig. 1b. Therefore, in order to meaningfully respond to global emission reduction targets, climate change mitigation programs should ideally aim to target all of the leading three GHGs—i.e., CO_2_, CH_4_ and N_2_O. A series of recently published disparate studies have evaluated the effectiveness of mineral-based technologies in mitigating each of the major GHGs. The following sections highlight the scope and degree of success of these investigations.

## Mineral GHG Mitigation Technologies: Applications and Effectiveness

### CO_2_ Sequestration

Mafic and ultramafic rocks, including basalt and ophiolitic rocks, are globally widespread and enriched in magnesium and iron silicates that are highly susceptible to chemical weathering according to the Goldich Series. Mafic mineral weathering involves the consumption of atmospheric carbon dioxide and generation of poorly-soluble carbonate precipitates and silicic acid. Equation 1 shows the weathering reaction of fayalite (the olivine Fe end-member) as an example, which weathers to the mineral siderite:

1 $${{\rm{Fe}}}_{2}{{\rm{SiO}}}_{4}+2{{\rm{CO}}}_{2}+2{{\rm{H}}}_{2}{\rm{O}}\leftrightarrow 2{{\rm{FeCO}}}_{3}+{{\rm{H}}}_{4}{{\rm{SiO}}}_{4}$$

The above process has been investigated for its potential as a negative emission technology (NET) across a range of applications from managing silicate-rich metallurgical waste (Jia et al. 2022) and mine tailings (Power et al. 2020) to supporting agricultural crops (Haque et al. 2019). In addition to promoting stabilisation of poorly-soluble carbonate precipitates, this NET approach can also lead to the transport of alkaline-rich phases to oceans offering further opportunity for atmospheric CO_2_ drawdown (Renforth 2012).

Outside of established industrial carbon capture and storage methods which have been widely reported on Kelemen et al. (2019) and Sanna et al. (2012), agriculture arguably offer the greatest impact for silicate weathering due to significant scale-up potential. Tropical landscapes in particular offer promise for the approach given accelerated weathering rates (Edwards et al. 2017). Moreover, application of mafic silicate minerals to agricultural landscapes offers additional potential benefits to climate change mitigation, including the supply of key crop nutrients (particularly calcium and magnesium) and soil acidification neutralisation. Several researchers have evaluated the viability of silicate weathering in agricultural settings (Edwards et al. 2017; Kantola et al. 2017; Andrews and Taylor 2019; Verbruggen et al. 2021). Reported offset effectiveness ranges from 0.2 to 0.8t CO_2_ t rock^−1^ (Edwards et al. 2017; Kantola et al. 2017) at application rates of ~10 t rock ha^−1^. However, several potential drawbacks to the approach are noted including increased sedimentation, leaching of toxic metals and an increased mining and processing footprint (Edwards et al. 2017).

### CH_4_ Abatement

Accounting for ~20% of anthropogenic GHG emissions across a diverse range of sources, CH_4_ is a key climate change contributor. Several minerals have shown CH_4_ mitigation potential. Iron hydroxides—particularly goethite and ferrihydrite—as well as sulfates—gypsum and barite—have shown promise in decreasing CH_4_ emissions from rice paddies, with up to 83% emission reductions observed (Jäckel and Schnell 2000; Denier van der Gon et al. 2001; Minamikawa and Sakai 2005; Huang et al. 2009). The basis for the approach is that the iron and sulfur in the added minerals are utilised by iron and sulfur-reducing bacteria, which outcompete methanogens in anaerobic soil environments encountered in rice farming landscapes. Effective application rates are generally in the range of 100–200 kg mineral ha^−1^ (Minamikawa and Sakai 2005), although Denier van der Gon et al. (2001) note that absolute emission reduction is highly location dependent. Given the importance of rice farming as a source of CH_4_ emissions (ca. 1.5 Pg CO_2_-e year^−1^, Sriphirom et al. 2020), a mineral-based mitigation approach could be effective especially factoring-in additional nutrient supply benefits (Ca, Mg, S and Fe) from the minerals.

Zeolites are another mineral group that can mitigate CH_4_ emissions. Their high surface area, particularly for synthetic zeolites, offers prospects for catalytic chemical CH_4_ oxidation (Jackson et al. 2019). Potential applications for catalytic chemical CH_4_ oxidation using zeolites include coal mine ventilation air, fugitive emissions from oil and gas pipework, as well as exhaust air from ruminant animal housing (Pratt and Tate 2018).

Minerals with high surface areas, including perlite and allophane, have also be harnessed to enhance biological CH_4_ oxidation for some key emission sources. Bacteria termed ‘methanotrophs’ consume CH_4_ as their sole substrate, producing water and CO_2_ which is a far less potent GHG than CH_4_. Methanotrophs are globally ubiquitous in soils, sediments and waterways and have been used in engineered filters and covers to eliminate CH_4_ in coal mines (Limbri et al. 2014), dairy farms (Syed et al. 2017) and landfills (Thomasen et al. 2019).

### Stable Soil C Sequestration

A recent and growing body of research has highlighted the importance of inorganic minerals in helping to protect soil organic carbon (Kirschbaum et al. 2020; Almeida et al. 2021). Mineral groups identified as playing key roles in carbon protection include clays and oxides/hydroxides (Gentsch et al. 2018). These minerals, sometimes referred to as ‘secondary’ minerals, are formed by the chemical and physical weathering of primary silicate minerals and consequently are typically very fine-grained with high surface areas.

The precise mechanisms of carbon protection offered by these minerals remain unclear, and it is important to note that they do not offer permanent ‘locking up’ of carbon in the soil environment, as even the most recalcitrant carbon compounds are ultimately accessible to microorganisms (Keiluweit et al. 2015; Kleber et al. 2021). Nonetheless, the presence of secondary minerals in soils does appear to result in enhanced carbon protection over short to long-term time frames (Dungait et al. 2019). Sarkar et al. (2018) note that carbon protection by soil secondary minerals is broadly a two-stage process, beginning with organic compounds becoming physically and chemically sorbed to the mineral via a host of mechanisms (e.g., electrostatic attraction, hydrophobic bonding, development of inner sphere complexes), and culminating in a decrease in the kinetics of the microbial turnover of these ‘protected’ compounds.

Despite inorganic minerals showing promise to protect soil carbon from atmospheric loss, several challenges currently limit its development into a fully formed negative emission technology. The first is uncertainty over the time frame of carbon protection, as discussed above. Then there is the matter of resolving the quantity of carbon shielded from atmospheric loss. This is obviously a difficult exercise, requiring compilation of enormous laboratory and field-scale datasets able to be accommodated into widely accepted predictive models.

Nonetheless, appraisal of global soil stocks highlights the significant climate change abatement potential of harnessing high surface area minerals. Bossio et al. (2020) suggest that soils can contribute ~24 Pg CO_2_-e year^−1^ in GHG mitigation potential via protection of existing soil carbon as well as ‘rebuilding depleted stocks’. It is important to note that this figure, whilst very high, was derived from land management-based methods—such as restoration, conservation and stock grazing rotation—rather than via direct application of minerals to soil. Nonetheless, it does offer an insight into the potential impact that a mineral-based mitigation approach could offer in the field of soil carbon protection.

Harnessing high surface area minerals to assist in soil carbon protection could be achieved via targeting the addition of organic amendments to high surface area soils, as proposed by Kirschbaum et al. (2020). Agricultural land would clearly be the primary target for harnessing high surface area minerals to protect soil carbon, and this sector has been previously noted for its substantial carbon sequestration potential (Rosenzweig and Tubiello 2007). However, soil carbon protection by minerals could also be extended to other land uses such as civic urban greenspaces and rehabilitated mine sites. Nevertheless, potential increased sedimentation due to introduction of fine particles in the application area, as well as the mining impacts at the source area still need to be properly assessed to ensure the environmental viability of the application.

### N_2_O and NH_3_ Mitigation

The nitrogen-based GHGs—i.e., N_2_O and NH_3_, which is an indirect GHG—are mostly associated with agricultural sources. Hill et al. (2016) reported that addition of clays (vermiculite) to soils amended with various N fertiliser forms resulted in a 70% decrease in N_2_O emissions in a glasshouse trial. The ratio of clay-to-nitrogen needed for effective N_2_O mitigation was very high: 150:1. In related work, Pratt et al. (2016) reported that addition of clays (vermiculite and montmorillonite) to soils amended with organic and inorganic N fertilisers decreased N_2_O and ammonia emissions by >50% in a laboratory incubation experiment, although at similarly high clay/N ratios.

Koenig et al. (2005) found that magnesium chloride (MgCl_2_) and gypsum (CaSO_4_) reduced NH_3_ emissions by 75% and 65% respectively in a simulated in-house poultry composting facility. The minerals were applied at a rate of 40 g per kg of manure. Lefcourt and Meisinger (2001) reported that zeolite addition to housed dairy slurry decreased NH_3_ volatilisation by 50% at a zeolite application rate of 6.25% relative to the slurry mass.

Sun et al. (2016) evaluated the potential for lignite (brown coal) to decrease N-based emissions from beef feed pens. They concluded that although the lignite application resulted in higher direct N_2_O emissions, the corresponding decrease in NH_3_ losses resulted in a net N_2_O decrease. However, the authors do not discuss the GHG emissions associated with the carbon content of the lignite itself. Direct CO_2_ emissions from the labile fraction (20%) of the lignite alone would more than double the observed nitrogen GHG reductions over a 1 year period—when considered on a balanced CO_2_-equivalent basis.

## Potential Impact of an Integrated Mineral-Based GHG Mitigation Approach

We quantified the potential impact of the various mineral-based GHG mitigation efforts discussed above using the primary sources’ emission reduction estimates in conjunction with Life Cycle Assessment of GHG footprint estimates reported by Moosdorf et al. (2014). A key assumption is that the footprint estimates comprehensively derived by Moosdorf et al. (2014) for extraction, preparation and transportation of mafic rocks to agricultural land are applicable to the other mineral application technologies discussed. In principle, this assumption is reasonable given that the study by Moosdorf et al. (2014) captured global average inputs for transport scenarios, which included distances between mineral source and application destination that were about as far apart as possible for points on the Earth’s surface. Moreover, the footprint values calculated by Moosdorf et al. (2014) were expressed on a tonne CO_2_ emission per tonne of rock/mineral needed basis.

One further key factor considered in quantifying global mineral GHG abatement potential was the importance of achieving at least one other benefit to climate change mitigation alone. This is a crucial practical consideration, as even businesses and governments with strong climate change abatement intent still foresee risk in committing to stand-alone GHG mitigation approaches. Consequently, only applications that present compelling additional benefits to climate change mitigation are included in the analysis here.

**Fig. 2:**
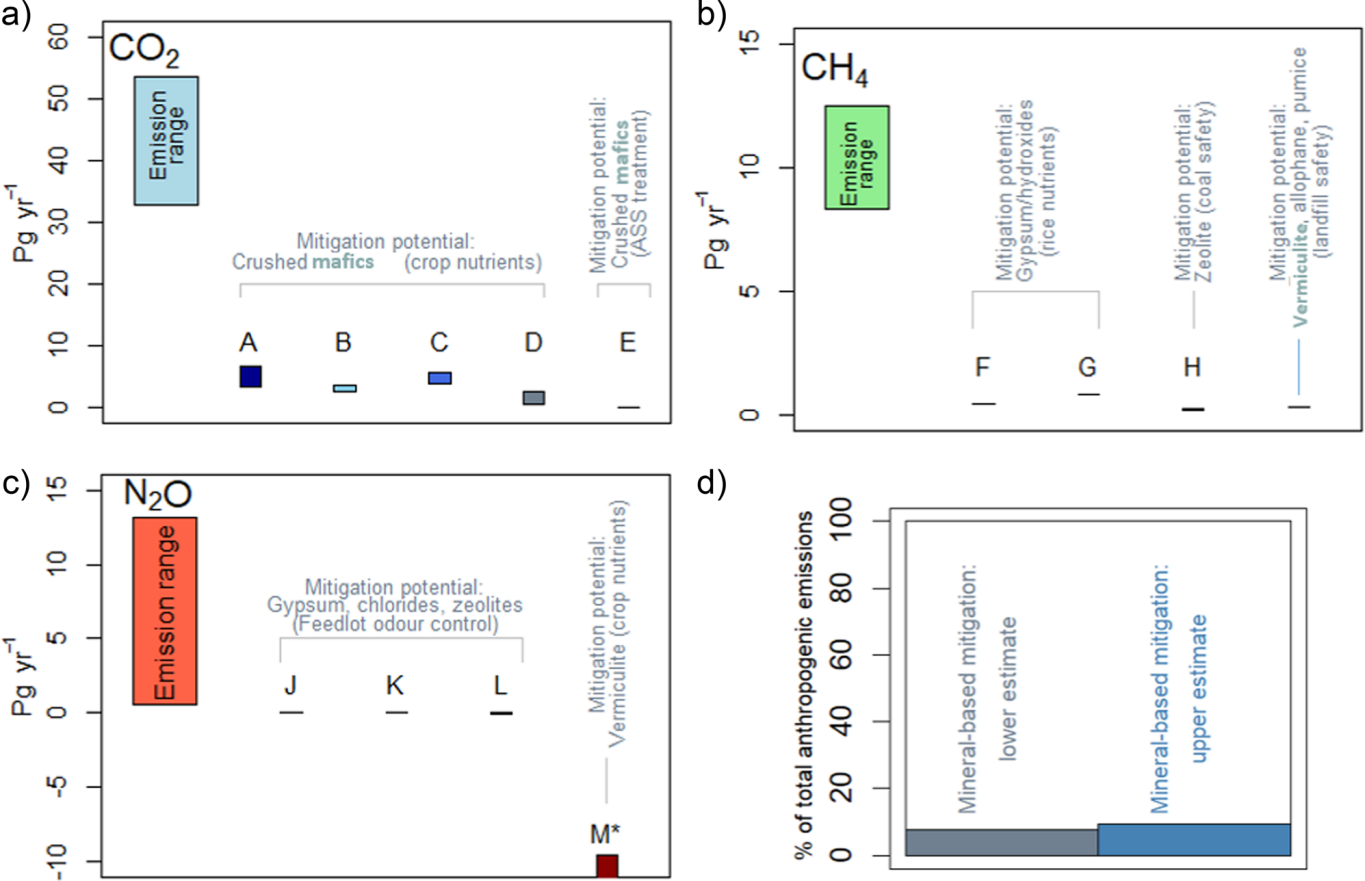
Mineral-based GHG mitigation estimate ranges for the ‘Big 3’ GHGs, (**a**) CO_2_, (**b**) CH_4_, (**c**) N_2_O, as well as (**d**) total percentage mineral-based mitigation potential for all gases combined. ASS acid sulfate soils. For input sources refer to Table 1. Ranges for Life Cycle Assessment reported by Moosdorf et al. (2014) used in calculating upper and lower emission mitigation estimates for all applications

The results are shown in Fig. 2. Details of the key inputs for the analysis are presented in Table 1. Mineral-based CO_2_ abatement offers the highest total mitigation potential, largely owing to the sheer magnitude of CO_2_ generation relative to the other GHGs (Fig. 2a). Potential application areas include cropping land, where addition of olivine-rich rocks offer nutrient supply as well as CO_2_ drawdown; and acid sulfate soils, where base silicates can buffer soil acidity.

**Table 1:** Input reference sources for estimates calculated in Fig. 2

Data point in Fig. 2	Primary reference reporting mitigation potential	References used to extrapolate scale-up potential
A. CO_2_—Crushed mafics application to tropical cropland	Moosdorf et al. (2014)	(1) Area of tropical cropping land: 680 million ha (Edwards et al. 2017)
	#Upper and lower carbon footprint estimates (involving mining, processing and transport) used from this source for all cases below	(2) Application rate of crushed mafics: 10 t ha^−1^ year^−1^ (Edwards et al. 2017)
		(3) GHG mitigation estimates extrapolated using primary reference’s values combined with (1) and (2) above
B. As above	Edwards et al. (2017)	(4) Primary reference used for area of tropical cropping land and application rate: see (1) and (2) above for values
		(5) GHG mitigation estimates derived from combining (4) with (1) and (2) and CO_2_ sequestration estimate (based on stoichiometry of mafics weathering reaction)
C. As above	Köhler et al. (2010)	(6) Primary reference used for application amounts and GHG mitigation estimate
D. As above	Strefler et al. (2018)	(7) Primary reference used for GHG mitigation estimate, conservatively scaled-down to more realistic land application rate (10 t ha^−1^ year^−1^)
E. CO_2_—Crushed mafics application to impacted acid sulfate soils	Shazana et al. (2013)	(8) Primary reference used for GHG mitigation estimate, as well as land application rate (4 t ha^−1^ year^−1^) to mitigate acidity, (9) Michael et al. (2016) for global distribution of disturbed acid sulfate soils (21 Mha)
F. CH_4_—Sulfate mineral application to rice farms	Lindau (1994)	(9) Primary reference used for GHG mitigation estimate and sulfate application rate (2 t ha^−1^ year^−1^), (10) Total CH_4_ emission from rice farming: 1 Gt CO_2_-e year^−1^ (Pratt and Tate 2018)
G. As above	Minamikawa and Sakai (2005)	(11) Primary reference used for GHG mitigation estimate, as well as land application rate (0.14 t ha^−1^ year^−1^, Total rice farm CH_4_ emission from (10) above
H. CH_4_—Zeolite application to ventilation air methane in coal mines	Thiruvenkatachari et al. (2009)	(10) For total CH_4_ emission from coal mining, (12) Karakurt et al. (2011) for proportion of CH_4_ from ventilation air and (13) Proportion of CH_4_ mitigatable by high surface area minerals from Thiruvenkatachari et al. (2009)
I. CH_4_—Pumice/Clays to landfill covers	Pratt et al. (2013)	(10) For total CH_4_ emission from landfills. (14) Abbasi (2018) for typical biogas recovery efficiency (40%), (15) IPCC (2014) for default landfill cover CH_4_ oxidation efficiency (10%)
J. NH_3_—Chlorides, livestock housing	Koenig et al. (2005)	(16) Total NH_3_ emissions from housed livestock from Beusen et al. (2008) assuming N_2_O emission factor of 0.1 from IPCC (2014)
K. NH_3_—Sulfates, livestock housing	Koenig et al. (2005)	As above
L. NH_3_—Zeolites, livestock housing	Lefcourt and Meisinger (2001)	As above
M. N_2_O—Clays, agricultural cropping	Hill et al. (2016)	(17) N_2_O emission rate per kg added N from Laubach et al. (2015), (18) Shcherbak et al. (2014) for total N_2_O emission across global cropland

Total mineral-based CH_4_ mitigation potential is lower than but proportionally similar to CO_2_ (Fig. 2b). Key application areas identified were rice farming, coal mines and landfills, with the mineral addition offering a broad suite of multi-benefits ranging from crop nutrient supply to hazardous and nuisance gas control.

Mineral-based N_2_O mitigation does not currently present a compelling opportunity (Fig. 2c)—the footprint emissions associated with the large quantities of mineral required are simply prohibitively high. In the study by Hill et al. (2016), a ratio of mineral:nitrogen of nearly 150:1 was needed to achieve effective mitigation. Future research would have to demonstrate much lower mineral requirements for promise in this application.

Combined mitigation estimates for CH_4_ and CO_2_ range between 9% and 11% of total anthropogenic GHG emissions (Fig. 2d)—or ~5 Gt CO_2_-e. This is an exceptionally high contribution and highlights the profound role that mineral-based technologies could play in climate control strategies.

## Availability of GHG-Mitigating Minerals

**Fig. 3:**
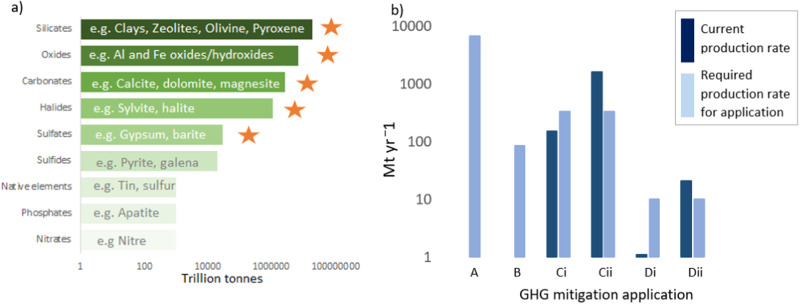
**a** Estimated crustal abundances of major mineral groups, computed from elemental and chemical datasets reported by Yaroshevsky (2006) and Faure (1998). Groups marked with stars possess substantive GHG mitigation potential as discussed in previous sections; **b** Current production volumes plotted against volumes that would be required to meet GHG mitigation application target: A = crushed mafics to tropical agricultural land, required volumes from Edwards et al. (2017); B = crushed mafics to acid sulfate soils, and required volumes from Shazana et al. (2013) and Michael et al. (2016); Ci = gypsum to rice farms, current production estimates from USGS (2022); Cii = Fe oxides to rice farms, current production estimates from USGS (2023a)—Lindau (1994) for required volumes for Ci and Cii; D = Clays to landfill covers, current production estimates from USGS (2023c)—Pratt et al. (2013) for required volumes for D

While minerals offer promise to mitigate GHG emissions across a range of sectors, abundance and supply are key considerations if mineral-based GHG mitigation technologies are to be pursued at scale. In Fig. 3a, the major mineral groups in the Earth’s crust are ranked according to abundance. The key groups with GHG mitigation potential are also the most abundant—all with crustal estimates exceeding 1000 trillion tonnes.

More important than crustal abundance, however, are accessible reserves and production quantities. Figure 3b presents these metrics for the most promising mineral groups and applications highlighted in Fig. 2 (note, the coal mine application is not included here as the effectiveness of inorganic minerals was only indirectly alluded to in that study). We attempted to estimate global production rates of crushed mafics, but it was too difficult to obtain accurate figures here. The USGS (2023b) estimates a production rate of 1.5 billion tonnes/year for general aggregate, but this is for the US only and does not distinguish the mafic component from other constituents. Cooper et al. (2018) estimate the global production of aggregate rocks to be >60 Gt/year but, again, it is not clear what fraction of this figure is apportioned to mafic rocks.

For the other rock minerals, it was possible to calculate global production rates (Fig. 3b). It is evident that the current mineral production rate for several applications would need to be escalated in pursuing a global mineral-based GHG initiative. Crustal abundances of key minerals suggest this could be achieved, however, the economic and environmental costs of such an initiative need to be considered. Crucially, it must be noted that current production feeds existing markets only. The redirection of any current production into new markets to support climate change mitigation will certainly cause significant disruption. Therefore, any proposed new markets will have to either find new production systems, or outcompete existing sources.

Nevertheless, increased demand may contribute to technological advancements in production methods which may unlock reserves that are currently inaccessible and lead to reduction of cost due to economies of scale. This has been observed in many natural resources sectors, most notably the fossil fuel industry.

## Minerals for GHG Mitigation: Challenges and Barriers

**Fig. 4:**
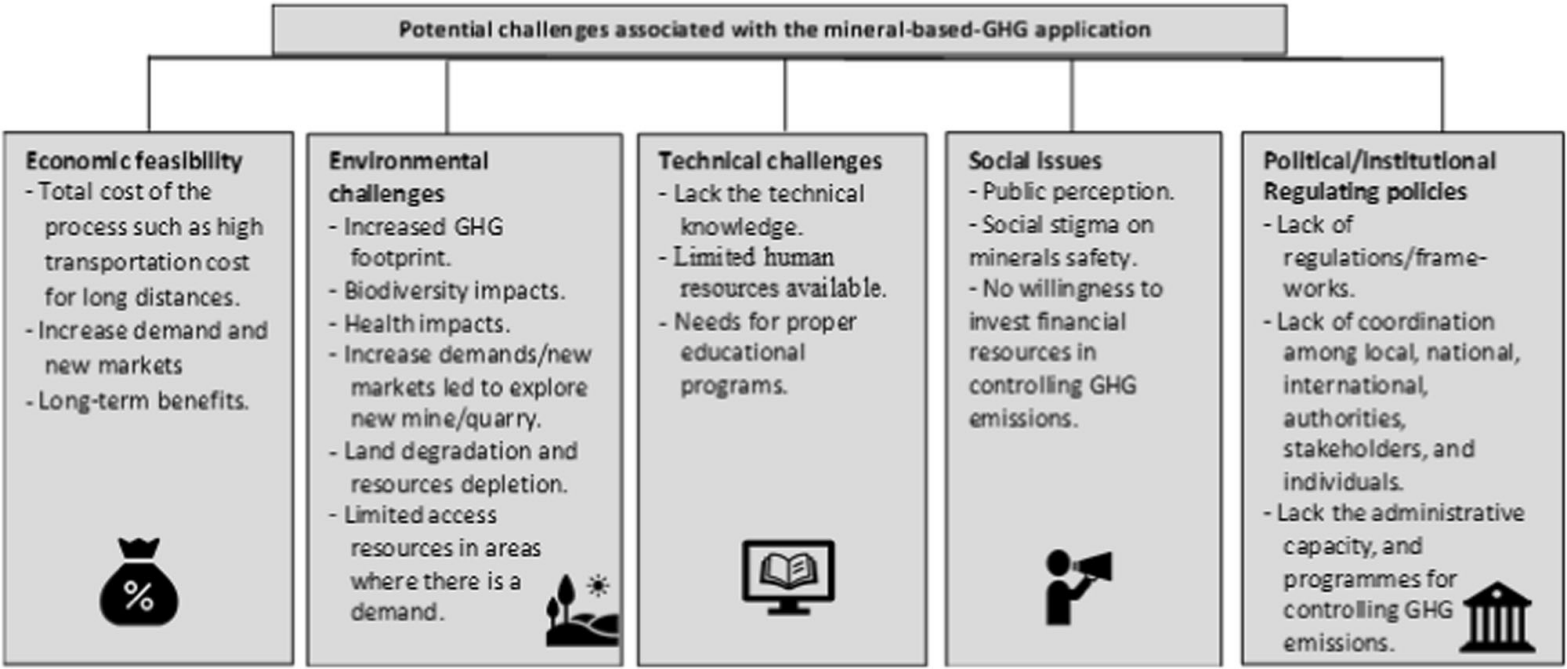
Potential challenges associated with the mineral-based GHG application

Several challenges and risks are associated with increased use of minerals to mitigate GHG emissions. These include: (1) technology cost, (2) increased GHG footprint related to mining, processing of minerals, storage and transportation, (3) leaching of harmful trace of metal ions, (4) biodiversity and biosphere–atmosphere impacts on connected fields, (5) resource depletion and land use degradation, (6) implication on receiving catchments’ water quality, and (7) health impacts on community (McLellan and Corder 2013; Edwards et al. 2017). Key aspects of these challenges are summarised in Fig. 4 and discussed below.

### Economic Perspectives

Accurate mineral-based GHG mitigation costs are difficult to establish. Although, the approach is generally perceived to be cost-effective (Strefler et al. 2018), a few studies have noted that high energy costs, high maintenance and operating costs and the need for qualified, fully trained and educated personnel present substantial inherent costs imbedded in with the technique (Pour et al. 2020).

Minerals that exhibit high GHG mitigation potential are globally abundant. However, their application towards a dedicated global GHG mitigation strategy would need a substantial increase in the production rates of minerals, thus extensive mining, crushing, grinding, transportation and proper storage (Strefler et al. 2018). Accessing sufficient resources in areas with a high demand presents a significant challenge. It is certain that with such high demands, new markets will open up to supply for the demands, which will promote new investment in exploration. However, there are formidable obstacles to face when constructing large scale mining sites, as they require substantial funding as well as resolving intricate social and environmental issues (Christmann 2018). Furthermore, the process from exploration to production of a new mine or quarry could be take about than 10 years to finalise depending on the regulatory framework (Shtiza 2020). In the study of Strefler et al. they found that more than 3 Gt of basalt is needed to be spread each year to sequester only 1 Gt CO_2_ per annum. Beerling et al. (2020) reported that the average costs of CO_2_ removal using rock weathering approach in USA is about US$160–180 per ton, while in European nations and Canada is US$160–190 per ton which is about 50% above than those in Mexico, India, Brazil, China, and Indonesia (US$55–120 t^−1^). The discrepancy is primarily affected by the cost of labour, energy requirements, and fuel costs. Bulk minerals, such as crushed rock, often face barriers and limitations due to the extremely expensive transportation costs. This impose a limitation on the bulk minerals to be confined to short transport distances of 50–60 km, challenging the notions of the worldwide market. In addition to cost, environmental benefits may diminish beyond these distances (Segura-Salazar and Tavares 2021).

Both crushing and grinding minerals are energy intensive (Koornneed and Nieuwlaar 2009). Crushing minerals to fine size is deemed a necessary step in order to achieve reaction rates on practical time scales. The needed grain size has a significant impact on the energy demand for grinding as shown in Table 2. The production of very fine particles (less than 10 µm) will require exceptionally high cost of energy. The relationship between total global costs of minerals based GHG, revenues, and profits as a function of particle size was illustrated by Strefler et al. (2018) who found that the carbon price must exceed the threshold (about 280 US$ t^−1^ CO_2_ removed) for profit.

**Table 2:** Data extracted from Strefler et al. (2018) using “https://apps.automeris.io/wpd/”

Costs for comminution energy							
to 2 μm	US$ t rock^−1^	to 10 μm	US$ t rock^−1^	to 20 μm	US$ t rock^−1^	to 50 μm	US$ t rock^−1^
Lower	27.779	Lower	3.6785	Lower	2.2714	Lower	1.4178
Median	72.985	Median	11.7968	Median	5.4080	Median	2.7093
Upper	197.000	Upper	39.6580	Upper	20.3522	Upper	8.7996

### Technical and Environmental Challenges

Comminution processes including grinding, crushing, and rock transportation from mines to fields represent the main contributors of the possible health and environmental impacts (Eufrasio et al. 2022). Rock dust is widely utilised as an acceptable fertiliser in agriculture to improve soil health and fertility (Beerling et al. 2020). However, the creation of fine dust particles can pose a serious risk, implying potential health risks from respiration. Fine particulate matter with particle size less than 10 µm is a major problem that can seriously affect human health causing human respiratory diseases (Strefler et al. 2018).

Land degradation and resource depletion associated with GHG emission from increased mineral extraction activities is another serious issue. Mining is a major contributor to natural land transformation and degradation, and resource depletion (Eufrasio et al. 2022).

Loss of vegetation cover is one of largest footprints. Large amounts of topsoil, which is rich in organic matter and contains the majority of the plant nutrients, are removed during mineral extraction, which has a negative impact on the physical, chemical, and biological characteristics of soil as well as plant growth (Worlanyo and Jiangfeng 2021).

Growing geological inputs into agricultural soils could be fraught with dangers including impacts on biodiversity and water quality as well as the potential for mineral and rock washout to contaminate waterways. Goll et al. (2021) reported that despite the positive impacts of using basalt dust for CO_2_ sequestration, the massive deployment of basalt dust was a major concern due to its negative impact on the biodiversity, biosphere–atmosphere, and on water, soil, and air pollution. They found that the quantity of basalt dust used, the ecosystem status, and the fate of phosphorus released from basalt dust are affecting river chemistry and biodiversity. Edwards et al. (2017) and Worlanyo and Jiangfeng (2021) stated that washing of silicates into water bodies during heavy rains and flooding events increases inorganic turbidity and sedimentation, reducing the breeding of river fish and other aquatic organisms as a result of the high concentration of bioavailable metals and metalloids. Thus, local fisheries and conservation can be adversely affected.

Another issue with using ultramafic minerals is the leaching of toxic metal ions, specifically Ni and Cr (Suhrhoff 2022). These ions are potentially toxic in soil at high concentrations. For example, in the study of Renforth et al. (2015), 99% of the Ni and Cr released from olivine weathering were held in the soil using agricultural soil core, limiting the addition of olivine to soils to 95 tonne per hectare before acceptable Ni concentrations are exceeded.

An application of silicate minerals can be used to reduce the risk of nitrate mobilisation (de Oliveira Garcia et al. 2020). However, their application to agricultural soils requires careful assessment as these minerals could potentially be problematic due to the release of metal ions and persistent organic compounds (Beerling et al. 2020).

Another challenge to be considered, especially in the case of agricultural land is the potential for crushed minerals to become a nutrient transport medium within run-off and dust (Rajmohan and Elango 2005). Balancing mineral soil inputs so that nutrient requirements are not exceeded can manage this problem and potentially decrease reliance on continued input of chemical fertilisers.

Finally, use of minerals to abate climate change will also entail social and political dilemmas (Kantzas et al. 2022). Nowadays, there is a wide debate regarding using more sustainable and green technologies for mineral and metal extraction with minimum environmental impacts, and re-use of by products and waste from mineral extraction to support transition to a circular economy. However, global CO_2_ removal success is highly dependent on overcoming the political and social issues in shaping of the process by setting up the necessary laws and regulations, proper frameworks and engaging the public to understand the potential benefits of the process (Beerling et al. 2020). Also, sufficient scientific understanding of the potential environmental and health impacts of the application and assuring the public of the safety and effectiveness of the method is essential. However, most of the mineral-based GHG practices are focused primarily in a region or a country where materials are to be used. Thus, public acceptance is crucial by involving local, national, international, political authorities, stakeholders, and individual farm scales to recognise the need and the benefits of the process.

Lack in operational skilled labour in the local area results in greater uncertainty in modelling CO_2_ emissions from production and to implement climate change mitigation practices (Pour et al. 2020). Thus, financial, industrial and regulating policy authorities need to work together on road mapping the possibility of short-term and long-term goals of the mineral-based GHG as a tool for climate risk mitigation.

## Conclusions

- Minerals offer substantial climate change mitigation potential for each of the ‘big 3’ greenhouse gases—i.e., carbon dioxide (CO_2_), methane (CH_4_) and nitrous oxide (N_2_O)—across a range of application sectors.
- Current practices reveal mineral-based N_2_O mitigation is not effective, due to prohibitively large mineral quantities required to abate emissions.
- By contrast, minerals can mitigate ~10% of global CO_2_ and CH_4_ emissions by targeting applications in the agriculture, land restoration and waste management sectors.
- The key minerals that could be used to achieve this level of abatement are globally abundant, although new pathways to production would need to be explored to support a dedicated climate change mitigation approach.
- Economic, environmental and social impacts of the approach need to be researched in detail.

## Data Availability

The datasets generated during and/or analysed during the current study are available from the corresponding author on reasonable request.

## References

[CR1] Abbasi S (2018). The myth and the reality of energy recovery from municipal solid waste. Energy Sustain Soc.

[CR2] Almeida LF, Souza IF, Hurtarte LC, Teixeira PPC, Inagaki TM, Silva IR, Mueller CW (2021). Forest litter constraints on the pathways controlling soil organic matter formation. Soil Biol Biochem.

[CR3] Andrews MG, Taylor LL (2019). Combating climate change through enhanced weathering of agricultural soils. Elem Int Mag Mineral Geochem Petrol.

[CR4] Bambace L, Ramos F, Lima I, Rosa R (2007). Mitigation and recovery of methane emissions from tropical hydroelectric dams. Energy.

[CR5] Beerling DJ, Kantzas EP, Lomas MR, Wade P, Eufrasio RM, Renforth P, Sarkar B, Andrews MG, James RH, Pearce CR (2020). Potential for large-scale CO_2_ removal via enhanced rock weathering with croplands. Nature.

[CR6] Beerling DJ, Kantzas EP, Lomas MR, Wade P, Eufrasio RM, Renforth P, Sarkar B, Andrews MG, James RH, Pearce CR (2020). Potential for large-scale CO2 removal via enhanced rock weathering with croplands. Nature.

[CR7] Beusen A, Bouwman A, Heuberger P, Van Drecht G, Van Der Hoek K (2008). Bottom-up uncertainty estimates of global ammonia emissions from global agricultural production systems. Atmos Environ.

[CR8] Bossio DA, Cook-Patton SC, Ellis PW, Fargione J, Sanderman J, Smith P, Wood S, Zomer RJ, von Unger M, Emmer IM, Griscom BW (2020). The role of soil carbon in natural climate solutions. Nat Sustain.

[CR9] Brauch MD, Touchette Y, Cosbey A, Gerasimchuk I, Sanchez L, Bernasconi-Osterwalder N, Garcia MBT, Potaskaevi T, Petrofsky E (2019) Treaty on sustainable investment for climate change mitigation and adaptation: aligning international investment law with the urgent need for climate change action. J Int Arbitr 36(1):7–35

[CR10] Charola AE, Pühringer J, Steiger M (2007). Gypsum: a review of its role in the deterioration of building materials. Environ Geol.

[CR11] Christmann P (2018). Towards a more equitable use of mineral resources. Nat Resour Res.

[CR12] Cooper AH, Brown TJ, Price SJ, Ford JR, Waters CN (2018). Humans are the most significant global geomorphological driving force of the 21st century. Anthr Rev.

[CR13] Crippa M, Solazzo E, Guizzardi D, Monforti-Ferrario F, Tubiello FN, Leip A (2021). Food systems are responsible for a third of global anthropogenic GHG emissions. Nat Food.

[CR14] Crowther TW, Todd-Brown KE, Rowe CW, Wieder WR, Carey JC, Machmuller MB, Snoek B, Fang S, Zhou G, Allison SD (2016). Quantifying global soil carbon losses in response to warming. Nature.

[CR15] Denier van der Gon HA, van Bodegom PM, Wassmann R, Lantin RS, Metra-Corton TM (2001). Sulfate-containing amendments to reduce methane emissions from rice fields: mechanisms, effectiveness and costs. Mitig Adapt Strateg Glob Change.

[CR16] Dungait J, Berhe AA, Gregory AS, Hopkins DW (2019) Physical protection and mean residence time of soil carbon. In: Lai R, Stewart BA (eds) Soil and climate. CRC Press, Roca Baton, p 171–181

[CR17] Edwards DP, Lim F, James RH, Pearce CR, Scholes J, Freckleton RP, Beerling DJ (2017). Climate change mitigation: potential benefits and pitfalls of enhanced rock weathering in tropical agriculture. Biol Lett.

[CR18] Eufrasio RM, Kantzas EP, Edwards NR, Holden PB, Pollitt H, Mercure J-F, Koh SCL, Beerling DJ (2022). Environmental and health impacts of atmospheric CO_2_ removal by enhanced rock weathering depend on nations’ energy mix. Commun Earth Environ.

[CR19] Faure G (1998) Principles and applications of geochemistry, 2nd edn. Prentice Hall, Upper Saddle River, NJ, USA

[CR20] Gentsch N, Wild B, Mikutta R, Čapek P, Diáková K, Schrumpf M, Turner S, Minnich C, Schaarschmidt F, Shibistova O (2018). Temperature response of permafrost soil carbon is attenuated by mineral protection. Glob Change Biol.

[CR21] Goll DS, Ciais P, Amann T, Buermann W, Chang J, Eker S, Hartmann J, Janssens I, Li W, Obersteiner M, Penuelas J, Tanaka K, Vicca S (2021). Potential CO_2_ removal from enhanced weathering by ecosystem responses to powdered rock. Nat Geosci.

[CR22] Haque F, Santos RM, Dutta A, Thimmanagari M, Chiang YW (2019). Co-benefits of wollastonite weathering in agriculture: CO_2_ sequestration and promoted plant growth. ACS Omega.

[CR23] Henriet O, Fourmentin J, Delincé B, Mahillon J (2014). Exploring the diversity of extremely halophilic archaea in food-grade salts. Int J Food Microbiol.

[CR24] Hill J, Redding M, Pratt C (2016). A novel and effective technology for mitigating nitrous oxide emissions from land-applied manures. Anim Prod Sci.

[CR25] Huang B, Yu K, Gambrell RP (2009). Effects of ferric iron reduction and regeneration on nitrous oxide and methane emissions in a rice soil. Chemosphere.

[CR26] IEA (2021) Greenhouse gas emissions from energy data explorer. https://www.iea.org/data-and-statistics/data-tools/greenhouse-gas-emissions-from-energy-data-explorer.

[CR27] IPCC (2014) Climate change 2014: synthesis report. IPCC, Geneva, Switzerland: Contribution of working groups I, II and III to the fifth assessment report of the Intergovernmental Panel on Climate Change [Core Writing Team, Pachauri RK, Meyer LA (eds)]. Intergovernmental Panel on Climate Change, Switzerland

[CR28] IPCC (2021) Climate change 2021: the physical science basis. Contribution of working group I to the sixth assessment report of the Intergovernmental Panel on Climate Change [Masson-Delmotte V, Zhai P, Pirani A, Connors SL, Péan C, Berger S, Caud N, Chen Y, Goldfarb L, Gomis MI, Huang M, Leitzell K, Lonnoy E, Matthews JBR, Maycock TK, Waterfield T, Yelekçi O, Yu R, Zhou B (eds)]. Cambridge University Press, Cambridge, United Kingdom and New York, NY, USA (In press). 10.1017/9781009157896.

[CR29] IPCC (2023) Summary for policymakers. In: Core Writing Team, Lee H, Romero J (eds) Climate change 2023: synthesis report. Contribution of Working Groups I, II and III to the sixth assessment report of the Intergovernmental Panel on Climate Change. IPCC, Geneva, Switzerland, p 1–34. 10.59327/IPCC/AR6-9789291691647.001

[CR30] Ireland P, Clausen D (2019) Local action that changes the world: fresh perspectives on climate change mitigation and adaptation from Australia. In: Letcher TM (ed) Managing global warming. Elsevier, London, UK, p 769–782

[CR31] Jäckel U, Schnell S (2000). Suppression of methane emission from rice paddies by ferric iron fertilization. Soil Biol Biochem.

[CR32] Jackson RB, Solomon E, Canadell J, Cargnello M, Field C (2019). Methane removal and atmospheric restoration. Nat Sustain.

[CR33] Jia X, Zhang Z, Wang F, Li Z, Wang Y, Aviso KB, Foo DY, Nair PNSB, Tan RR, Wang F (2022). Regional carbon drawdown with enhanced weathering of non-hazardous industrial wastes. Resour Conserv Recycl.

[CR34] Kantola IB, Masters MD, Beerling DJ, Long SP, DeLucia EH (2017). Potential of global croplands and bioenergy crops for climate change mitigation through deployment for enhanced weathering. Biol Lett.

[CR35] Kantzas EP, Val Martin M, Lomas MR, Eufrasio RM, Renforth P, Lewis AL, Taylor LL, Mecure J-F, Pollitt H, Vercoulen PV, Vakilifard N, Holden PB, Edwards NR, Koh L, Pidgeon NF, Banwart SA, Beerling DJ (2022). Substantial carbon drawdown potential from enhanced rock weathering in the United Kingdom. Nat Geosci.

[CR36] Karakurt I, Aydin G, Aydiner K (2011). Mine ventilation air methane as a sustainable energy source. Renew Sustain Energy Rev.

[CR37] Keiluweit M, Bougoure JJ, Nico PS, Pett-Ridge J, Weber PK, Kleber M (2015). Mineral protection of soil carbon counteracted by root exudates. Nat Clim Change.

[CR38] Kelemen P, Benson SM, Pilorgé H, Psarras P, Wilcox J (2019). An overview of the status and challenges of CO2 storage in minerals and geological formations. Front Clim.

[CR39] Kirschbaum MU, Moinet GY, Hedley CB, Beare MH, McNally SR (2020). A conceptual model of carbon stabilisation based on patterns observed in different soils. Soil Biol Biochem.

[CR40] Kleber M, Bourg IC, Coward EK, Hansel CM, Myneni SCB, Nunan N (2021). Dynamic interactions at the mineral–organic matter interface. Nat Rev Earth Environ.

[CR41] Koenig RT, Palmer MD, Miner FD, Miller BE, Harrison JD (2005). Chemical amendments and process controls to reduce ammonia volatilization during in-house composting. Compost Sci Util.

[CR42] Köhler P, Hartmann J, Wolf-Gladrow DA (2010). Geoengineering potential of artificially enhanced silicate weathering of olivine. Proc Natl Acad Sci.

[CR43] Koornneed J, Nieuwlaar E (2009) Environmental life cycle assessment of CO_2_ sequestration through enhanced weathering of olivine. Working paper, Group Science, Technology and Society, Utrecht University

[CR44] Laubach J, Heubeck S, Pratt C, Woodward K, Guieysse B, Van Der Weerden T, Chung M, Shilton A, Craggs R (2015). Review of greenhouse gas emissions from the storage and land application of farm dairy effluent. NZ J Agric Res.

[CR45] Lefcourt A, Meisinger J (2001). Effect of adding alum or zeolite to dairy slurry on ammonia volatilization and chemical composition. J Dairy Sci.

[CR46] Lima IB, Ramos FM, Bambace LA, Rosa RR (2008). Methane emissions from large dams as renewable energy resources: a developing nation perspective. Mitig Adapt Strateg Glob Change.

[CR47] Limbri H, Gunawan C, Thomas T, Smith A, Scott J, Rosche B (2014). Coal-packed methane biofilter for mitigation of green house gas emissions from coal mine ventilation air. PLoS ONE.

[CR48] Lindau C (1994). Methane emissions from Louisiana rice fields amended with nitrogen fertilizers. Soil Biol Biochem.

[CR49] McLellan B, Corder G (2013). Risk reduction through early assessment and integration of sustainability in design in the minerals industry. J Clean Prod.

[CR50] Michael P, Fitzpatrick R, Reid R (2016). The importance of soil carbon and nitrogen for amelioration of acid sulphate soils. Soil Use Manag.

[CR51] Minamikawa K, Sakai N (2005). The effect of water management based on soil redox potential on methane emission from two kinds of paddy soils in Japan. Agric Ecosyst Environ.

[CR52] Moosdorf N, Renforth P, Hartmann J (2014). Carbon dioxide efficiency of terrestrial enhanced weathering. Environ Sci Technol.

[CR53] Neumann RB, Moorberg CJ, Lundquist JD, Turner JC, Waldrop MP, McFarland JW, Euskirchen ES, Edgar CW, Turetsky MR (2019). Warming effects of spring rainfall increase methane emissions from thawing permafrost. Geophys Res Lett.

[CR54] de Oliveira Garcia W, Amann T, Hartmann J, Karstens K, Popp A, Boysen LR, Smith P, Goll D (2020). Impacts of enhanced weathering on biomass production for negative emission technologies and soil hydrology. Biogeosciences.

[CR55] Pangala SR, Reay DS, Heal KV (2010). Mitigation of methane emissions from constructed farm wetlands. Chemosphere.

[CR56] Pour SH, Abd Wahab AK, Shahid S, Asaduzzaman M, Dewan A (2020). Low impact development techniques to mitigate the impacts of climate-change-induced urban floods: current trends, issues and challenges. Sustain Cities Soc.

[CR57] Power IM, Dipple GM, Bradshaw PM, Harrison AL (2020). Prospects for CO_2_ mineralization and enhanced weathering of ultramafic mine tailings from the Baptiste nickel deposit in British Columbia, Canada. Int J Greenh Gas Control.

[CR58] Pratt C, Tate K (2018). Mitigating methane: emerging technologies to combat climate change’s second leading contributor. Environ Sci Technol.

[CR59] Pratt C, Walcroft AS, Deslippe J, Tate KR (2013). CH_4_/CO_2_ ratios indicate highly efficient methane oxidation by a pumice landfill cover-soil. Waste Manag.

[CR60] Pratt C, Redding M, Hill J, Brown G, Westermann M (2016). Clays can decrease gaseous nutrient losses from soil‐applied livestock manures. J Environ Qual.

[CR61] Rajmohan N, Elango L (2005). Nutrient chemistry of groundwater in an intensively irrigated region of southern India. Environ Geol.

[CR62] Renforth P (2012). The potential of enhanced weathering in the UK. Int J Greenh Gas Control.

[CR63] Renforth P, Pogge von Strandmann PAE, Henderson GM (2015). The dissolution of olivine added to soil: Implications for enhanced weathering. Appl Geochem.

[CR64] Ritchie H, Roser M, Rosado P (2020) CO_2_ and greenhouse gas emissions. Our World in Data, Oxford, UK

[CR65] Rosenzweig C, Tubiello FN (2007). Adaptation and mitigation strategies in agriculture: an analysis of potential synergies. Mitig Adapt Strateg Glob Change.

[CR66] Sanna A, Hall MR, Maroto-Valer M (2012). Post-processing pathways in carbon capture and storage by mineral carbonation (CCSM) towards the introduction of carbon neutral materials. Energy Environ Sci.

[CR67] Sarkar B, Singh M, Mandal S, Churchman GJ, Bolan NS (2018) Clay minerals—organic matter interactions in relation to carbon stabilization in soils. In: Garcia C, Nannipieri P, Hernandez T (eds) The future of soil carbon. Elsevier, London, UK, p 71–86

[CR68] Segura-Salazar J, Tavares LM (2021). A life cycle-based, sustainability-driven innovation approach in the minerals industry: Application to a large-scale granitic quarry in Rio de Janeiro. Miner Eng.

[CR69] Shazana M, Shamshuddin J, Fauziah C, Syed Omar S (2013). Alleviating the infertility of an acid sulphate soil by using ground basalt with or without lime and organic fertilizer under submerged conditions. Land Degrad Dev.

[CR70] Shcherbak I, Millar N, Robertson GP (2014). Global metaanalysis of the nonlinear response of soil nitrous oxide (N_2_O) emissions to fertilizer nitrogen. Proc Natl Acad Sci.

[CR71] Shtiza A (2020) Access to raw materials: industrial minerals prospective & EU policy. MINATURA

[CR72] Sriphirom P, Chidthaisong A, Yagi K, Tripetchkul S, Towprayoon S (2020). Evaluation of biochar applications combined with alternate wetting and drying (AWD) water management in rice field as a methane mitigation option for farmers’ adoption. Soil Sci Plant Nutr.

[CR73] Strefler J, Amann T, Bauer N, Kriegler E, Hartmann J (2018). Potential and costs of carbon dioxide removal by enhanced weathering of rocks. Environ Res Lett.

[CR74] Strefler J, Amann T, Bauer N, Kriegler E, Hartmann J (2018). Potential and costs of carbon dioxide removal by enhanced weathering of rocks. Environ Res Lett.

[CR75] Suhrhoff TJ (2022) Phytoprevention of heavy metal contamination from terrestrial enhanced weathering: can plants save the day? Front Clim 3:82020

[CR76] Sun J, Bai M, Shen J, Griffith DW, Denmead OT, Hill J, Lam SK, Mosier AR, Chen D (2016). Effects of lignite application on ammonia and nitrous oxide emissions from cattle pens. Sci Total Environ.

[CR77] Syed R, Saggar S, Tate K, Rehm BH, Berben P (2017). Assessing the performance of floating biofilters for oxidation of methane from dairy effluent ponds. J Environ Qual.

[CR78] Taylor LL, Quirk J, Thorley RM, Kharecha PA, Hansen J, Ridgwell A, Lomas MR, Banwart SA, Beerling DJ (2016). Enhanced weathering strategies for stabilizing climate and averting ocean acidification. Nat Clim Change.

[CR79] Thiruvenkatachari R, Su S, Yu XX (2009). Carbon fibre composite for ventilation air methane (VAM) capture. J Hazard Mater.

[CR80] Thomasen TB, Scheutz C, Kjeldsen P (2019). Treatment of landfill gas with low methane content by biocover systems. Waste Manag.

[CR81] Turner PA, Griffis TJ, Lee X, Baker JM, Venterea RT, Wood JD (2015). Indirect nitrous oxide emissions from streams within the US Corn Belt scale with stream order. Proc Natl Acad Sci.

[CR82] USGS (2022) USGS Gypsum Statistics and Information, from chrome-extension://efaidnbmnnnibpcajpcglclefindmkaj; https://pubs.usgs.gov/periodicals/mcs2022/mcs2022-gypsum.pdf

[CR83] USGS (2023a) USGS Iron Ore Statistics and Information, from chrome-extension://efaidnbmnnnibpcajpcglclefindmkaj; https://pubs.usgs.gov/periodicals/mcs2023/mcs2023-iron-ore.pdf

[CR84] USGS (2023b) USGS Crushed Stone Statistics and Information, from chrome-extension://efaidnbmnnnibpcajpcglclefindmkaj; https://pubs.usgs.gov/periodicals/mcs2023/mcs2023-stone-crushed.pdf

[CR85] USGS (2023c) USGS Kaolinite (Clay Minerals) Statistics and Information, from chrome-extension://efaidnbmnnnibpcajpcglclefindmkaj; https://pubs.usgs.gov/periodicals/mcs2023/mcs2023-clays.pdf

[CR86] Verbruggen E, Struyf E, Vicca S (2021). Can arbuscular mycorrhizal fungi speed up carbon sequestration by enhanced weathering?. Plants People Planet.

[CR87] Walter Anthony K, Daanen R, Anthony P, Schneider von Deimling T, Ping C-L, Chanton JP, Grosse G (2016). Methane emissions proportional to permafrost carbon thawed in Arctic lakes since the 1950s. Nat Geosci.

[CR88] Whitney DL, Evans BW (2010). Abbreviations for names of rock-forming minerals. Am Mineral.

[CR89] Worlanyo AS, Jiangfeng L (2021). Evaluating the environmental and economic impact of mining for post-mined land restoration and land-use: a review. J Environ Manag.

[CR90] Wu P-C, Chen P-F, Do TH, Hsieh Y-H, Ma C-H, Ha TD, Wu K-H, Wang Y-J, Li H-B, Chen Y-C, Juang J-Y, Yu P, Eng LM, Chang C-F, Chiu P-W, Tjeng LH, Chu Y-H (2016). Heteroepitaxy of Fe_3_O_4_/muscovite: a new perspective for flexible spintronics. ACS Appl Mater Interfaces.

[CR91] Yaroshevsky A (2006). Abundances of chemical elements in the Earth’s crust. Geochem Int.

[CR92] Zaman M, Nguyen ML (2010). Effect of lime or zeolite on N2O and N2 emissions from a pastoral soil treated with urine or nitrate-N fertilizer under field conditions. Agric Ecosyst Environ.

